# Study of the mechanism of color change of prehnite after heat treatment

**DOI:** 10.1039/d2ra00318j

**Published:** 2022-01-24

**Authors:** Qianqian Wang, Qingfeng Guo, Niu Li, Li Cui, Libing Liao

**Affiliations:** School of Gemmology, China University of Geosciences Beijing 100083 China qfguo@cugb.edu.cn clayl@cugb.edu.cn; Beijing Key Laboratory of Materials Utilization of Nonmetallic Minerals and Solid Wastes, National Laboratory of Mineral Materials. School of Materials Sciences and Technology, China University of Geosciences Beijing 100083 China

## Abstract

The most common color of prehnite is green, while yellow prehnite is rare and precious. Heat treatment is usually an effective way to improve the color of gemstones, but whether heat treatment can improve the color of prehnite remains to be explored. In this paper, yellow-green prehnite samples were heat-treated under oxidizing and reducing atmospheres, and the composition, structure and chromogenic mechanism of the prehnite samples before and after the heat treatment were analyzed and summarized by means of X-ray Fluorescence Spectroscopy (XRF), X-ray diffractomer (XRD), *in situ* high temperature XRD, Fourier Transform Infrared Spectroscopy (FTIR), Micro-Raman Spectroscopy, UV-Vis Spectroscopy, and X-ray photoelectron spectroscopy (XPS). The results show that the change of the relative content and occupation position of Fe^2+^ and Fe^3+^ is the main reason for the color change of yellow-green prehnite. When the yellow-green prehnite is heated to 800 °C, in an oxidizing atmosphere, some of the Fe^2+^ is oxidized to Fe^3+^, the content of Fe^3+^ increases, and the color becomes brownish yellow; in a reducing atmosphere, some of the Fe^3+^ is reduced to Fe^2+^, the content of Fe^2+^ increases, and the color becomes grayish white. The UV-Vis absorption spectra of the oxidized and reduced samples at this temperature further showed that the absorption broadband at 520–700 nm caused by the charge transfer between Fe^2+^ and Fe^3+^ disappeared, resulting in a great change in the color of the prehnite. Our experimental model provides ideas and experimental data for the further study of prehnite heat treatment.

## Abstract 2

Mechanism of color change of prehnite after heat treatment.

## Graphical abstract

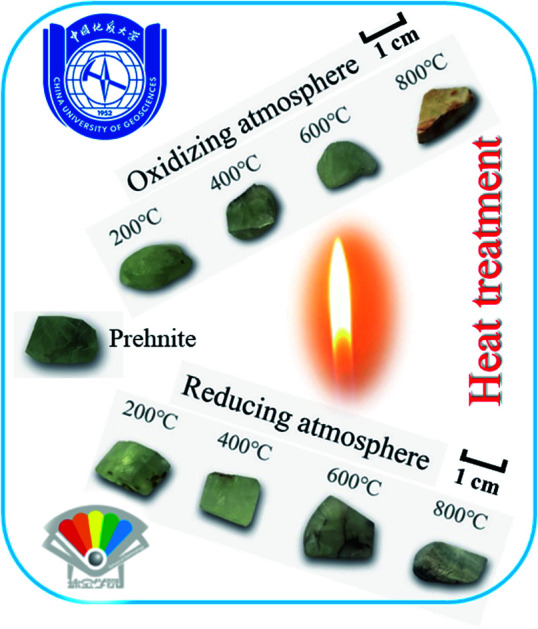

## 1. Introduction

Prehnite is a special layer-frame hydrous silicate mineral.^1^ It has attracted much attention because of its characteristic color. Prehnite often exists in magmatic rocks, sedimentary rocks and metamorphic rocks as a primary or secondary mineral, and its shapes are mostly botryoidal, radial or massive aggregates.^2–4^

Prehnite belongs to the orthorhombic system, and its molecular formula is Ca_2_Al[AlSi_3_O_10_](OH)_2_. Fe, Mg, Mn, Na, K and other elements can exist in the crystal structure of prehnite in the form of isomorphic substitution.^5,6^ The space group of prehnite was originally referred to as *Pncm* type by Peng.^1^ Akizuki^7^ used scanning electron microscopy and transmission electron microscopy to determine that the prehnite in Glasgow, Scotland was composed of polymorphous crystals with *Pncm*, *P*2*cm*, and *P*2/*n* symmetry.^8^ In 1967, Papike *et al.*^9,10^ found that the main features of the prehnite structures in the space group *Pncm* and *P*2*cm* are exactly the same. The main difference is that the order degree of tetrahedral silicon and aluminum is higher in the space group *P*2*cm* than in the space group *Pncm*. The distribution of silicon and aluminum in the tetrahedron determines the type of space group of prehnite.^11^ In addition, some scholars believe that the valence and occupancy of iron in the prehnite crystal also have a significant impact on its structure.^12^ Nagashima *et al.*^11^ found that Fe^3+^ usually replaces Al^3+^ in the octahedral coordination position in prehnite. Because Fe^3+^ has a larger radius than Al^3+^, the crystal lattice of prehnite is enlarged, and the structural parameters and single bonds are extended. The interplanar crystal spacing increases with the increase of Fe^3+^ content.^12,13^

The color of prehnite differs depending on the type of isomorphism replacement ions contained in it. The main chromogenic element of prehnite is Fe, and different content and position in the prehnite crystal lattice will affect the color of prehnite. Fe^2+^ and Fe^3+^ can exist in prehnite at the same time, and the relative content for Fe^2+^ and Fe^3+^ will also affect the color of the prehnite. In general, there are several mechanisms for heat treatment to change the color of gemstones, such as changing the valence state of coloring ions,^14^ destroying the original color center of gemstones,^15^ causing dehydration of some watery gemstones,^16^ diffusing the coloring ions in the gemstone,^17^ changing the lattice structure.^18^ It remains to be explored whether the color of prehnite can be improved by changing the valence state of the chromic element Fe through heat treatment. In 2019, Jing-yao *et al.*^19^ found that when prehnite was heat treated, the yellow tone of the prehnite color will be enhanced in the oxidizing atmosphere, while white tone of the prehnite color becomes deeper in the reducing atmosphere. However, in this study, the mechanism of the color change of prehnite after heat treatment was not discussed in detail, and the suitable heat treatment temperature range of prehnite still needs further discussion.

Based on this, the prehnite samples were placed in the oxidation atmosphere and the reduction atmosphere respectively, and uses *in situ* high-temperature XRD to find the appropriate heat treatment temperature. The changes of prehnite composition, structure and spectral characteristics were further studied by X-ray Fluorescence Spectroscopy (XRF), powder X-ray diffraction (XRD), Fourier Transform infrared spectroscopy (FTIR), laser Micro-Raman spectroscopy and UV-Vis absorption spectroscopy (UV-Vis), combined with X-ray photoelectron spectroscopy (XPS) to explore the effect of Fe ions on the color of prehnite. In order to obtain the suitable heat treatment temperature of prehnite and a series of change characteristics after heat treatment, verify whether heat treatment can improve the color of prehnite and obtain more information about the color change mechanism of prehnite after heat treatment, and enrich the experimental data of prehnite after heat treatment.

## 2. Materials and methods

### 2.1 Samples preparation

In this paper, a large prehnite from Africa was cut into 19 samples for experiments. The heat treatment experiments of the prehnite samples were carried out in the oxidizing atmosphere and the reducing atmosphere. The oxidizing atmosphere was carried out in air. The reducing atmosphere adopted the double crucible method and used carbon powder as the reducing agent. The samples were heated in the muffle furnace at a heating rate of 5 °C min^−1^ and heated to 200, 400, 600 and 800 °C respectively. The heat treatment experiments adopted the controlled variable method, and the conditions were uniformly set to constant temperature for 2 h and then cooled to room temperature.

### 2.2 Test parameters

The instrument used for the *in situ* high-temperature XRD test is the D8-Discover X-ray diffractometer produced by Bruker manufacturer in Germany. The test conditions are as follows, the test range is 5–90°, the scanning rate is 4° min^−1^, the heating rate is 10°/min, and each test temperature stays at a constant temperature for 5 minutes.

The instrument used for energy dispersive X-ray fluorescence (XRF) test is EDX-7000 energy dispersive X-ray Fluorescence Spectrometer produced by Shimadzu manufacturer in Japan. The experimental test conditions are: element analysis range is ^(11)^Na–^(92)^U, Al–U test voltage is 50 kV, Na–Sc test voltage is 15 kV, and the test environment is vacuumized before the test.

The instrument used for powder X-ray diffraction (XRD) is Dmax12 kW powder diffractometer. Experimental test conditions: Cu target, Kα radiation source (*λ* = 0.15418 nm), graphite curved crystal monochromator, tube voltage 40 kV, tube current 100 mA, the divergence slit and the scattering slit on the goniometer are both 1°, the scanning speed is 4° min^−1^, and the sampling step width is 0.02° (2*θ*), the scanning range is 5–80°.

The instrument used for the infrared spectroscopy test is the Tensor 27 Fourier infrared spectrometer produced by Bruker manufacturer in Germany. The transmission method is adopted for the test. The experimental test conditions are that the test voltage is 220 V, the test is performed at room temperature, the resolution is 4 cm^−1^, the background scanning time is 32 scans, the sample scanning time is 32 scans, the scanning speed is 10 kHz, and the scanning range is 4000–400 cm^−1^.

The instrument used for the Micro-Raman spectrum test is the HR-Evolution Micro-Raman spectrometer produced by Horiba manufacturer in Japan. Experimental test conditions: the test range is 4000–100 cm^−1^, the integration time is 3 s, the integration times is 1, the excitation light source is 532 nm, and the grating is 600 (500 nm).

The instrument used for the UV-Vis spectrum test is the UV-3600 model UV-Vis spectrophotometer produced by Shimadzu manufacturing plant in Japan. The detection method adopts the reflection method. The experimental test conditions: the test range is 200–900 nm, the light source conversion wavelength is 300 nm, the grating conversion wavelength is 850 nm, the detector conversion wavelength is 850 nm, the sampling interval is 0.5 s, and the dual beam mode is adopted.

The instrument used for XPS is ESCALAB 250Xi X-ray photoelectron spectrometer of Thermo Scientific. Instrument test parameters: the ion source is 100–4000 eV, the beam spot diameter is 1–10 mm, the sputtering rate range is 0.1–50 nm min^−1^, the metal sampling depth is 0.5–2 nm, the inorganic sampling depth is 1–3 nm, and the organic sampling depth is 3–10 nm.

## 3. Results and discussion

### 3.1 General properties

The conventional gemological properties of prehnite are tested. The refractive index of prehnite is 1.63 (points), and the density range is 2.80–2.95 g cm^−3^. Under different atmospheres, the density of prehnite gradually decreases with increasing temperature (Table 1). Prehnite shows fluorescence inertness under ultraviolet fluorescent lamps, and the fluorescence does not change obviously with the change of temperature.

**Table 1:** Density variation of prehnite under different conditions

Condition	Sample number	Density (g cm^−3^)
Oxidizing atmosphere	OA200-J	2.90
	OA400-J	2.85
	OA600-J	2.82
	OA800-J	2.76
Reducing atmosphere	RA200-J	2.90
	RA400-J	2.89
	RA600-J	2.87
	RA800-J	2.70

**Fig. 1:**
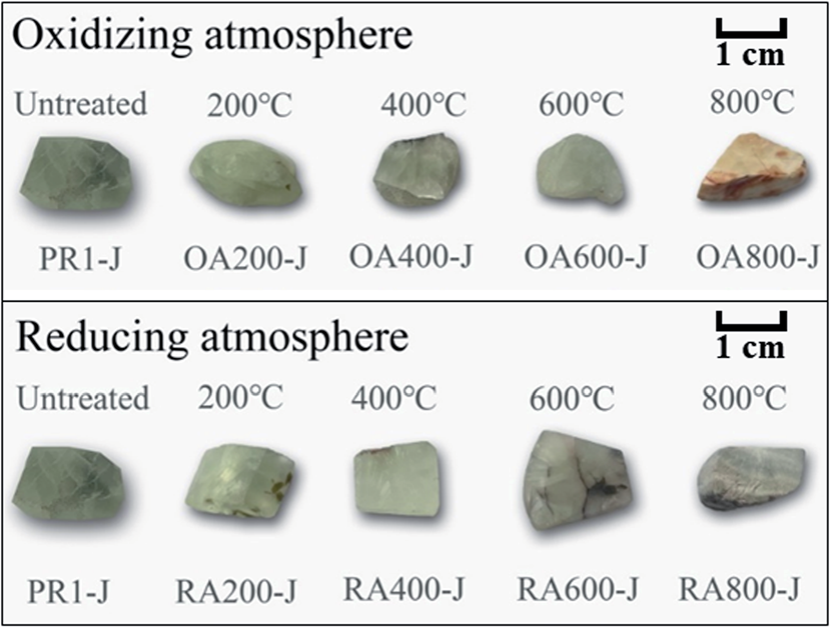
Untreated and heat treated prehnite samples.

The color changes of untreated and heat-treated prehnite samples are shown in Fig. 1. When untreated, all samples are uniformly yellow-green. In an oxidizing atmosphere, with the increase of temperature, the transparency of samples OA200-J, OA400-J, OA600-J and OA800-J gradually weakens, and the yellow tone gradually deepens. At 800 °C, the sample OA800-J turns brownish yellow. In a reducing atmosphere, the transparency of samples RA200-J, RA400-J, RA600-J, and RA800-J gradually decreases with increasing temperature, and the white tone gradually deepens. At 800 °C, the color of sample RA800-J changes from yellowish-green tone to nearly white tone.

### 3.2 *In situ* high-temperature XRD

**Fig. 2:**
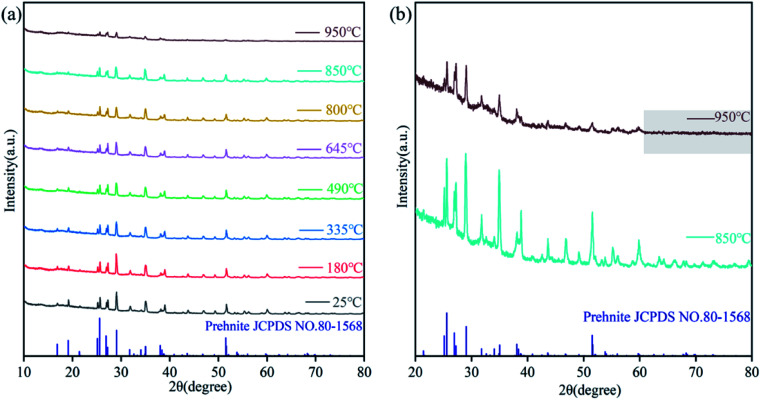
*In situ* high temperature XRD patterns of prehnite in the range of 25–950 °C. (a) 2*θ* = 10–80° XRD patterns; (b) 2*θ* = 20–80° XRD patterns at 850, 950 °C.

Prehnite is formed under low-temperature hydrothermal conditions, and high temperature will partially decompose it into other minerals. Previous studies have shown that new phases of prehnite will be formed when heated to 1073 K.^20^ In order to verify whether the experimental results are the same as those of previous studies and to explore the appropriate heat treatment temperature for prehnite, 25, 180, 335, 490, 645, 800, 850 and 950 °C were used as the experimental temperature of *in situ* high temperature XRD. Fig. 2 shows the *in situ* high temperature XRD patterns of prehnite sample at different temperatures under room temperature. Fig. 2(a) shows that at 25 °C, all the diffraction peaks of the sample were consistent with that of the standard card for prehnite (JCPDS no.80-1568) without any other impurities. When the temperature reaches 950 °C, the diffraction peaks of the sample disappear after 60°, and the intensity of some diffraction peaks is significantly weaker than that of 850 °C (Fig. 2(b)). When the temperature is 850 °C, the diffraction peaks of the prehnite sample did not change significantly compared with that of room temperature. Therefore, in order to better conduct the heat treatment experiment, the heat treatment temperature of the prehnite is controlled below 850 °C.

### 3.3 X-ray fluorescence spectroscopy (XRF)

Table 2 shows that the main constituent elements of the prehnite samples are Ca, Al, Si, in which the content range of SiO_2_ is 44.80–46.06%, the content range of CaO is 26.43–27.47%, and the content range of Al_2_O_3_ is 24.57–25.90%. The main chromogenic element of prehnite is Fe, which exists in the prehnite as a trace element, and the content of Fe_2_O_3_ in the sample ranges from 1.00 to 1.70%. In prehnite, Fe^3+^ often occupy the site for Al^3+^ with octahedral coordination, and Fe^2+^ replaces Ca^2+^ to occupy the lattice voids. In the oxidizing atmosphere, because part of Fe^2+^ is oxidized to Fe^3+^, the relative content of Fe^2+^ and Fe^3+^ changes. Therefore, the change of the relative content of CaO and Fe_2_O_3_ is selected to characterize the change of the relative content of Fe elements in different valence states. As shown in Table 2, the Fe_2_O_3_ content of the sample OA200-J is 1.00%, and the Fe_2_O_3_ content of the samples OA400-J, OA600-J and OA800-J are 1.59, 1.47 and 1.30% respectively, which are higher than that of the sample OA200-J. At this time, the CaO content of samples OA400-J, OA600-J, and OA800-J are 26.76, 27.05 and 26.99% respectively, which are also higher than that of sample OA200-J (26.68%). This is because part of Fe^2+^ is oxidized to Fe^3+^ in the oxidizing atmosphere, resulting in the reduction of Fe^2+^ content, and the isomorphic transformation between Fe^2+^ and Ca^2+^ is also reduced accordingly. When some Fe^3+^ and Ca^2+^ are isomorphic, in order to balance the electricity price, more Ca^2+^ is required during isomorphic transformation, so the CaO content is relatively high. The Fe_2_O_3_ content (1.59%) of sample OA400-J is higher than that of the sample OA800-J (1.30%), but the CaO content (26.76%) is lower than that of sample OA800-J (26.99%). The reason is that the sample is heated to 800 °C in oxidizing atmosphere, the degree of oxidation is greater, and Fe^2+^ is more oxidized to Fe^3+^, which affects the CaO content.

**Table 2:** XRF test results of samples before and after heat treatment

Name	SiO_2_	CaO	Al_2_O_3_	Fe_2_O_3_	SO_3_	V_2_O_5_	K_2_O	CuO
	Wt%							
PR1-J	45.466	27.470	24.802	1.000	0.933	0.185	0.097	0.018
OA200-J	45.884	26.682	25.380	1.003	0.794	0.141	0.105	0.011
OA400-J	44.801	26.760	25.605	1.593	0.969	0.151	0.110	0.010
OA600-J	45.822	27.052	24.569	1.472	0.855	0.091	0.119	0.007
OA800-J	45.134	26.987	25.293	1.299	0.961	0.099	0.192	0.011
RA200-J	45.417	26.616	25.897	1.102	0.786	0.085	0.073	0.016
RA400-J	46.062	26.426	24.889	1.697	0.832	0.042	0.035	0.011
RA600-J	45.669	26.566	25.523	1.314	0.770	0.059	0.052	0.024
RA800-J	45.164	27.149	25.416	1.173	0.839	0.111	0.064	0.014

Similarly, in the reducing atmosphere, because part of Fe^3+^ is reduced to Fe^2+^, the change of the relative content of Al_2_O_3_ and Fe_2_O_3_ is selected to characterize the change of the relative content of different valence Fe elements. The Fe_2_O_3_ contents of samples RA400-J, RA600-J, and RA800-J are 1.70, 1.31 and 1.17% respectively, which are also higher than that of sample RA200-J (1.10%), but the Al_2_O_3_ contents of samples RA400-J, RA600-J, and RA800-J are 24.89, 25.52 and 25.42% respectively, which are lower than that of sample RA200-J (25.90%). This is because in the reducing atmosphere, part of Fe^3+^ is reduced to Fe^2+^, resulting in the reduction of Fe^3+^ content, part of Fe^2+^ replaces Fe^3+^ to occupy octahedral coordination. When Fe^2+^ and Al^3+^ are isomorphic replaced, less Al^3+^ is required to balance the electricity price, the relative content of Al_2_O_3_ is low. Therefore, the change of the relative content of Fe^2+^ and Fe^3+^ is one of the reasons for the change of sample color.

### 3.4 X-ray diffraction (XRD)

**Fig. 3:**
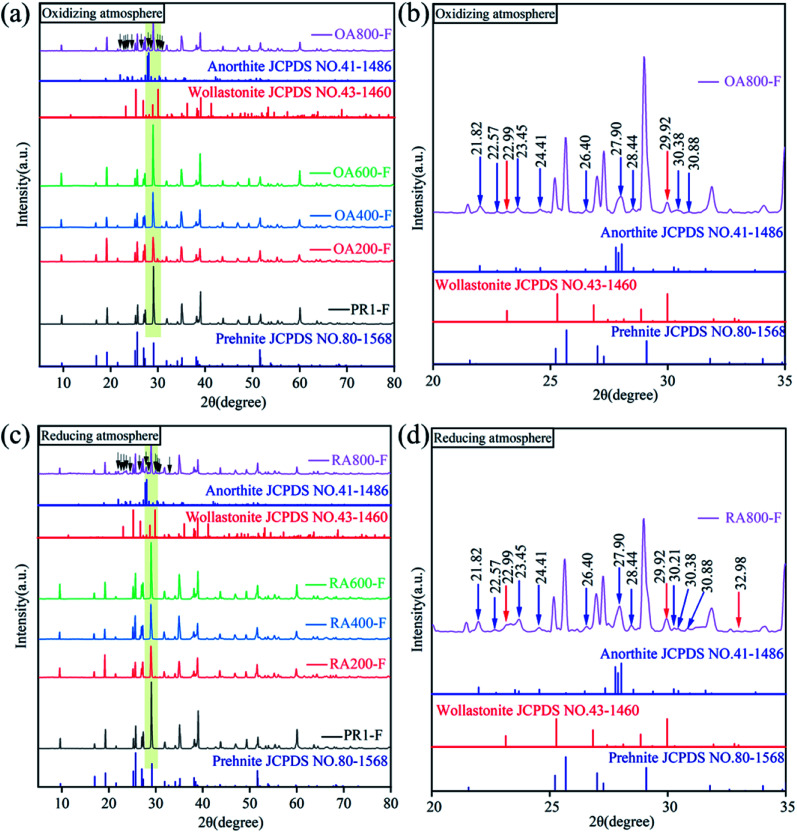
XRD patterns of yellow green prehnite samples after heat treatment in oxidizing atmosphere and reducing atmosphere. (a) XRD patterns in the range of 5–80° in the oxidizing atmosphere; (b) XRD pattern of sample OA800-F in the range of 20–35° in the oxidizing atmosphere; (c) XRD patterns in the range of 5–80° in the reducing atmosphere; (d) XRD pattern of sample RA800-F in the range of 20–35° in the reducing atmosphere.

The XRD patterns of samples heat treated at different temperatures under oxidizing and reducing atmospheres are shown in Fig. 3. The diffraction peaks of all samples indicate that the crystallinity of the samples is good. In addition, all the diffraction peaks for the samples can be well matched with that of standard card for prehnite (JCPDS no. 80-1568), indicating all the sample are pure prehnite phase. When the heating temperature reaches 800 °C, new diffraction peaks appear. As shown in Fig. 3(b) and (e), the diffraction peaks at 21.82, 22.57, 23.45, 24.41, 26.40, 27.90, 28.44, 30.38, and 30.88° (blue arrow) for sample OA800-F and sample RA800-F are consistent with the standard card of anorthite (JCPDS no. 41-1486), and the diffraction peaks at 22.99, 29.92° (red arrow) are consistent with the standard card of wollastonite (JCPDS no. 43-1460). However, diffraction peaks also appeared at 30.21° (blue arrow) and 32.98° (red arrow) of sample RA800-F in reducing atmosphere, while these two diffraction peaks did not appear in sample OA800-F in oxidizing atmosphere, indicating that the prehnite sample decomposed at 800 °C, and part of the prehnite is decomposed into anorthite and wollastonite, and the decomposition degree is greater in a reducing atmosphere than in an oxidizing atmosphere. Compared with 800 °C heat treatment, the *in situ* high temperature XRD at 800 °C of the sample was not decomposed into other minerals. The reason is that too short heating time and constant temperature retention time in the *in situ* high-temperature XRD is not enough to make the decomposition reaction.

### 3.5 Fourier transform infrared spectroscopy (FTIR)

**Fig. 4:**
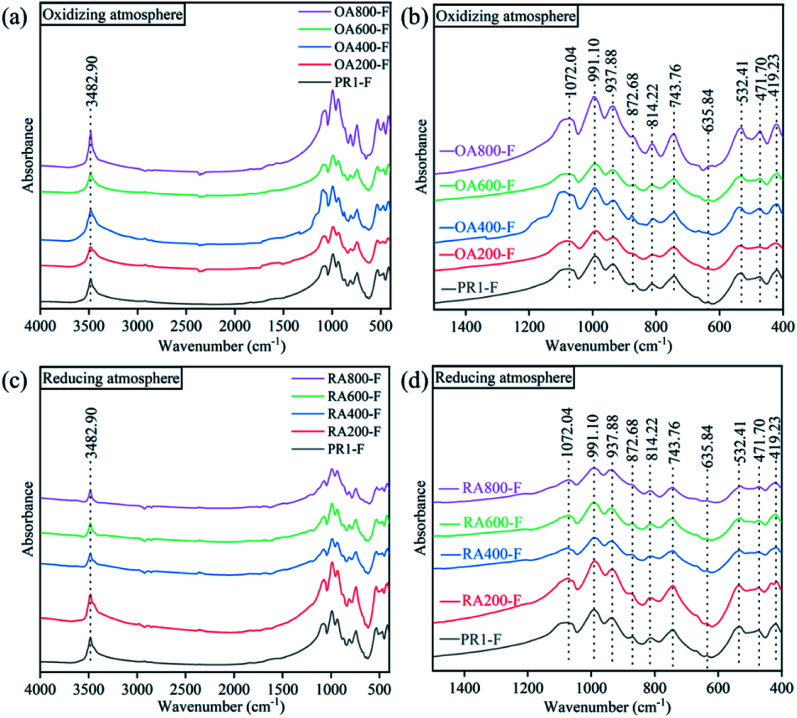
Infrared transmission spectra of heat treatment at different temperatures in oxidizing atmosphere and reducing atmosphere of prehnite sample. (a) 4000–400 cm^−1^ infrared transmission spectra of oxidation atmosphere; (b) 1500–400 cm^−1^ infrared transmission spectra of oxidation atmosphere; (c) 4000–400 cm^−1^ infrared transmission spectra of reducing atmosphere; (d) 1500–400 cm^−1^ infrared transmission spectra of reducing atmosphere.

The infrared spectra of the samples were depicted in Fig. 4. The untreated prehnite sample (PR1-F) has similar spectral characteristics to the heat-treated prehnite samples with characteristic peaks at 3483, 1072, 991, 938, 873, 814, 744, 636, 532, 472, and 419 cm^−1^. And with the increase of temperature, there is no obvious difference in the infrared transmission spectra of the samples under oxidizing atmosphere and reducing atmosphere. The characteristic peak at 3483 cm^−1^ is attributed to the OH stretching vibrations of prehnite,^21,22^ indicating the presence of constitution water in the sample. The absorption peak at 3483 cm^−1^ did not disappear after heat treatment of the sample, indicating that heat treatment of the sample under oxidizing atmosphere and reducing atmosphere below 800 °C will not cause the prehnite to completely lose constitution water. The absorption peak at 900–1100 cm^−1^ is related to Al^3+^–O–Si stretching vibration, while the absorption peak at 500–600 cm^−1^ is caused by the bending vibration of Si–O–A1^3+^ and the vibration of partially superposed MO_6_ octahedral groups. The absorption peak at 814 cm^−1^ is attributed to the bending vibration of the group H–O–Fe^3+^.^23^ In the reducing atmosphere, the intensity of the 814 cm^−1^ absorption peak gradually weakened with increasing temperature and gradually increased in the oxidizing atmosphere. This is due to the increase of Fe^3+^ content in the oxidizing atmosphere, and the strengthening of the bending vibration of H–O–Fe^3+^. The absorption peak of 472 cm^−1^ is related to the internal vibration mode of the MO_6_ octahedron.^21^ These characteristic peaks reflect that prehnite contains two framework silicon oxygen tetrahedron and aluminum oxygen octahedron.^24^

### 3.6 Micro-Raman spectroscopy

**Fig. 5:**
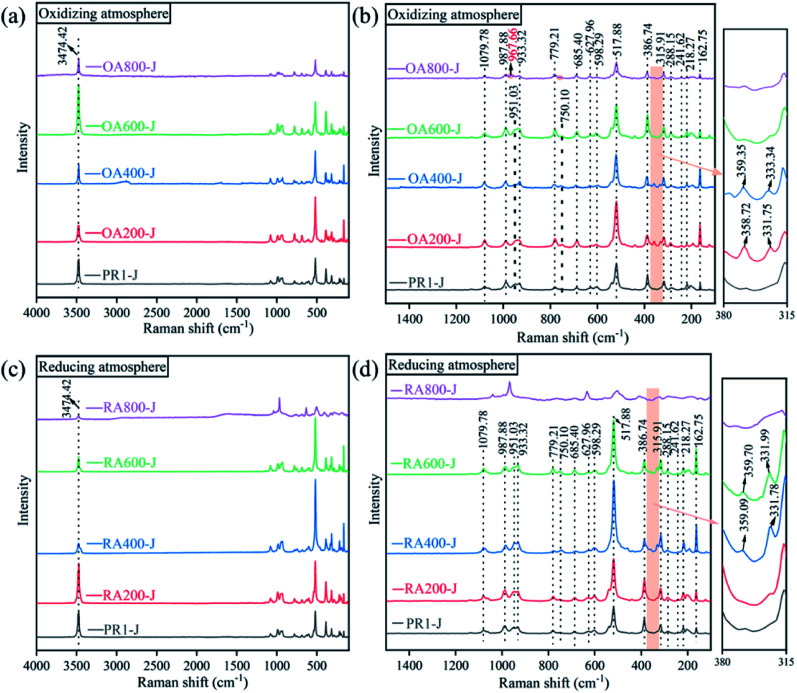
Raman spectra of heat treatment at different temperatures in oxidizing atmosphere and reducing atmosphere of prehnite sample. (a) 4000–100 cm^−1^ Raman spectra of oxidation atmosphere; (b) 1500–100 cm^−1^ Raman spectra of oxidation atmosphere; (c) 4000–100 cm^−1^ Raman spectra of reducing atmosphere; (d) 1500–100 cm^−1^ Raman spectra of reducing atmosphere.

Select points on the smoother surface of the prehnite sample for the Raman spectrum test. Fig. 5 shows the Raman spectra of the samples. The characteristic Raman shifts of the untreated prehnite samples (PR1-J) are mainly located at 3474, 1080, 988, 951, 933, 779, 750, 685, 628, 598, 518, 387, 316, 288, 242, 218, and 163 cm^−1^. The characteristic peak at 3474 cm^−1^ is ascribed to the OH stretching vibration, which indicates the presence of constitution water in prehnite. When the temperature reaches 800 °C, the characteristic peaks caused by OH stretching vibration still exist in both oxidizing atmosphere (OA800-J) and reducing atmosphere (RA800-J), indicating that when the heating temperature is 800 °C, the constitution water of prehnite does not completely lost. This is consistent with the previous results of infrared transmission spectra of the samples. Theoretical research shows that *Pncm*-type prehnite exhibits 60 Raman active vibration modes.^21,25^ In the Raman spectra shown in Fig. 5, only about 20 Raman peaks of prehnite samples can be saw in the range of 100–1500 cm^−1^. These Raman peaks can be divided into three groups, among which the characteristic peaks in the range of 850–1200 cm^−1^ are related to the symmetrical stretching mode and symmetrical bending mode of T–O and T–O–T (T = Al, Si). The characteristic peak at 1080 cm^−1^ belongs to the symmetric bending mode and symmetric stretching mode of the Si–O bond, while the characteristic peak in the range of 900–1000 cm^−1^ belongs to the vibration mode of the Al–O bond. The characteristic peaks in the range of 350–850 cm^−1^ are caused by metal cation stretching/bending and octahedral motion. The characteristic peaks in the range of less than 350 cm^−1^ belong to lattice modes.^20,21,25,26^

As the temperature increases, the Raman spectra intensity of the prehnite samples at the low frequency end (380–315 cm^−1^) firstly increases and moves to the high frequency end, and then gradually weakens or even disappears. In the oxidizing atmosphere, the Raman spectra at 380–315 cm^−1^ appears at 200 °C (OA200-J), weakens or even disappears at 600 °C (OA600-J), while in the reducing atmosphere, the Raman spectra at 380–315 cm^−1^ appears at 400 °C (RA400-J) and weakens or even disappears at 800 °C (RA800-J). The characteristic peaks in the range of 358–360 cm^−1^ are corresponded to metal cation vibration and octahedral vibration. In the oxidizing atmosphere, the Fe^2+^ in the lattice channel is oxidized to Fe^3+^, and the increase of Fe^3+^ content can make more Fe^3+^ replace Al^3+^ to occupy the octahedral position, resulting in the change of the vibration mode and the peak change. Part of the Fe^3+^ in the octahedral position is reduced to Fe^2+^ in the reducing atmosphere, and Fe^2+^ enters the octahedral lattice to change the vibration mode and cause the peak change. The increase of temperature can increase the molecular collision rate and widen the Raman peak. When prehnite were heated at 800 °C in the oxidizing atmosphere, the characteristic peak at 750 cm^−1^ for prehnite disappears (OA800-J), and the characteristic peak at 951 cm^−1^ moves to 968 cm^−1^. While the Raman spectrum of sample RA800-J changes significantly in the reducing atmosphere. Combined with the XRD pattern, the change of Raman spectra at 800 °C is due to the high-temperature decomposition reaction of prehnite.

### 3.7 UV-Vis spectroscopy

**Fig. 6:**
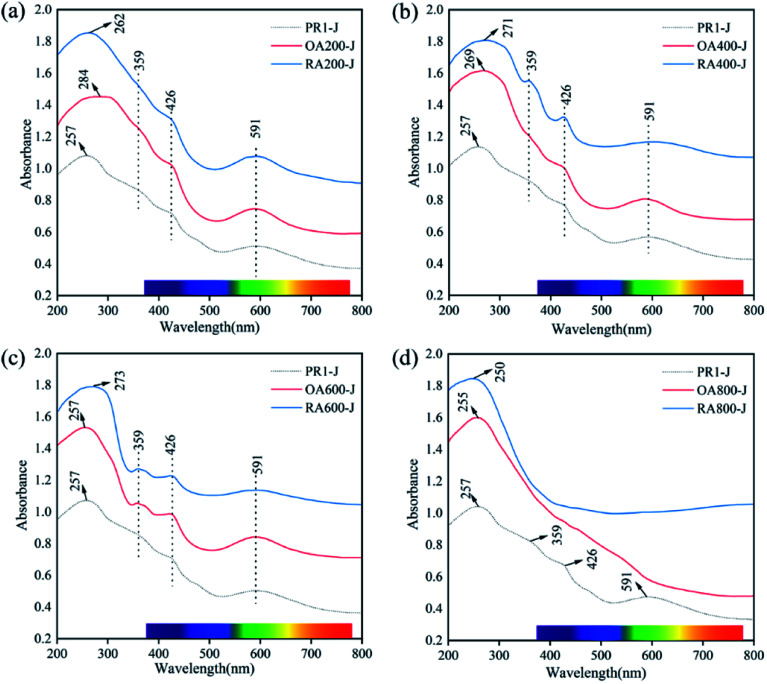
Representative UV-Vis absorption spectra of natural (point spectrum) and heat treatment in oxidizing atmosphere (red spectrum) and reducing atmosphere (blue spectrum) of prehnite samples. (a) The untreated sample is compared with the samples under oxidation atmosphere and reduction atmosphere at 200 °C; (b) comparison of samples at 400 °C; (c) comparison of samples at 600 °C; (d) comparison of samples at 800 °C.

The UV-Vis absorption spectra of the prehnite samples after heat treatment at different temperatures are shown in Fig. 6. All prehnite samples have a wide absorption band in the range of 200–320 nm, and the width of the absorption band gradually narrows with the increase of heating temperature. Unheated prehnite sample (PR1-J) has obvious broad bands in the range of 200–320 nm (centered at 257 nm), 320–395 nm (centered at 359 nm), 395–450 nm (centered at 426 nm), and 520–730 nm (centered at 591 nm). Below 600 °C, the absorption band of the heat-treated prehnite samples in the reducing atmosphere and the oxidizing atmosphere are located at about 359, 426, and 591 nm. When the heating temperature is 800 °C, the absorption peaks of samples OA800-J and RA800-J disappear at 359, 426, and 591 nm. The wavelength range of ultraviolet-visible light is 380–780 nm, so the change of the absorption broadbands at 426 and 591 nm is the main reason for the change of prehnite color.

The absorption broad band in the range of 200–320 nm is caused by the charge transfer of O^2−^ → Fe^3+^,^23^ and the absorption broad band ranging from 520 nm to 730 nm is due to the charge transfer between Fe^2+^–Fe^3+^. The absorption broad band near 359 nm is produced by the spin forbidden transition of Fe^3+^ d–d orbit (^6^A_1g_ → ^4^T_2g_).^27^ The absorption band around 426 nm indicates that the sample has Fe_6_^3+^ forbidden transition, and its absorption band belongs to the d–d orbit spin forbidden transition of Fe^3+^ (^6^A_1g_ → ^4^E_g_ + ^4^A_1g_).^28–30^ The yellow-green color of natural prehnite is attributed to the spin-forbidden transition of Fe^3+^ d–d orbital (^6^A_1g_ → ^4^E_g_ + ^4^A_1g_) and the charge transfer between Fe_ch_^2+^ and Fe_oct_^3+^. The change of Fe^2+^ and Fe^3+^ content and their occupancy positions are the main reasons for the changes of the absorption broadband of the sample after heat treatment.

**Fig. 7:**
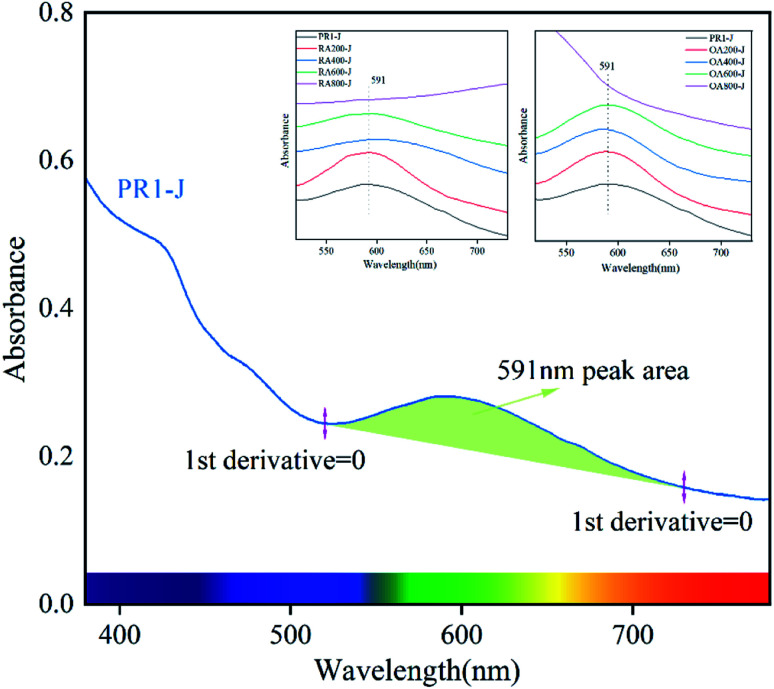
The UV-Vis spectrum of sample PR1-J at 380–780 nm and the peak area at 591 nm (start at 520 nm and end at 730 nm).

The change of the absorption band near 591 nm plays an important role in the change of prehnite color, and the area for the peak is calculated by ORIGIN software. Taking sample PR1-J as an example, select the point with zero first derivative near the 591 nm absorption peak as the starting point (520 nm) and the end point (730 nm) of the absorption broad band, and calculate the peak area under this broad band (Fig. 7 and Table 3).^31^ The results show that when the heating temperature is below 600 °C, the peak area of the absorption broad band gradually decreases in the range of 520–730 nm in the reducing atmosphere, and the peak area of the absorption broad band first decreases and then increases in the oxidizing atmosphere. At 800 °C, the absorption broad band ranging from 520 nm to 730 nm for OA800-J and RA800-J samples disappear. This is because when Fe^3+^ is reduced to Fe^2+^ in the reducing atmosphere, Fe^2+^ enters the octahedral position and the ion spacing between Fe^3+^ and Fe^2+^ changes. The 591 nm absorption peak previously caused by charge transfer between Fe_ch_^2+^ and Fe_oct_^3+^ now superimposes charge transfer between Fe_oct_^2+^ and Fe_oct_^3+^, and the change of the ratio Fe^3+^ to Fe^2+^ and distance between Fe^3+^ and Fe^2+^ makes the charge transfer rate different. While Fe^2+^ was oxidized to Fe^3+^ in the oxidizing atmosphere, and this kind of Fe^3+^ (Fe^2+^ → Fe^3+^) is present in the lattice channel. At this time, the 591 nm absorption peak is caused by the charge transfer of Fe_ch_^2+^ and Fe_oct_^3+^ superimposes the charge transfer of Fe_ch_^2+^ and Fe_ch_^3+^.

**Table 3:** Peak area of prehnite samples in range of 520–730 nm under different conditions

Condition	Sample number	Peak area
Oxidizing atmosphere	OA200-J	8.8885
	OA400-J	6.7225
	OA600-J	9.10475
	OA800-J	0
Reducing atmosphere	RA200-J	9.795
	RA400-J	6.109
	RA600-J	4.99075
	RA800-J	0

### 3.8 X-ray photoelectron spectroscopy (XPS)

**Fig. 8:**
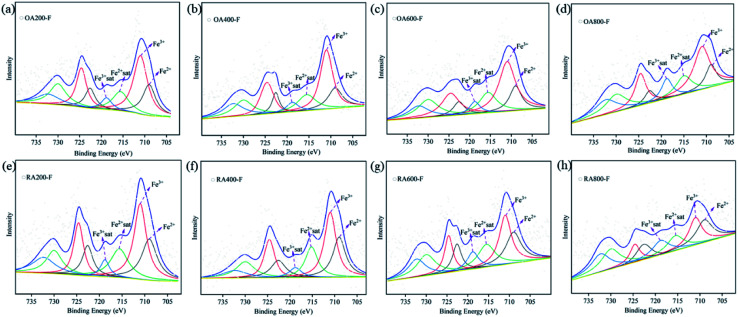
Shirley background-subtracted XPS spectra of Fe in prehnite samples at different temperatures in oxidizing atmosphere (a–d) and reducing atmosphere (e–h). Black, red, green and blue peaks are assigned Fe^2+^, Fe^3+^, satellite Fe^2+^ and satellite Fe^3+^ respectively.

In prehnite, Fe^3+^ often replaces Al^3+^ to occupy the octahedral coordination position, and Fe^2+^ replaces Ca^2+^ into the lattice channel. In this paper, XPS is used to analyze the valence state of Fe in prehnite samples, and the results are shown in Fig. 8. The C 1s binding energy of organic pollution carbon (284.8 eV) was used for charging calibration, and XPSPEAK was used for peak separation processing of the Fe data. The XPS results of the samples showed that Fe^2+^ and Fe^3+^ exist simultaneously in all prehnite samples, and the existing form is Fe 2p. The peak for Fe 2p can be divided into two peak positions, Fe 2p_1/2_ and Fe 2p_3/2_, and Fe 2p_3/2_ has a larger peak area than Fe 2p_1/2_. Previous studies have shown that in the Fe 2p_3/2_ region, the binding energy of Fe^3+^ is in the range of 710.3–712.6 eV, and that of Fe^2+^ is in the range of 708.9–709.0 eV. In the Fe 2p_1/2_ region, the binding energy of Fe^3+^ ranges from 724.4 to 724.8 eV, and that of Fe^2+^ ranges from 722.5 to 722.7 eV. Both Fe 2p_3/2_ and Fe 2p_1/2_ have associated satellite peaks.^32,33^Table 4 shows that the peak positions of the prehnite samples appear at 709.0, 711.0, 715.6, 718.8, 722.6, 724.6, 730.0, and 732.3 eV. In accordance with previous research results, the binding energies at 709.0 and 711.0 eV in the all the samples correspond to Fe^2+^ 2p_3/2_ and Fe^3+^ 2p_3/2_, respectively; the binding energies at 715.6 and 718.8 eV are satellite peaks of Fe^2+^ 2p_3/2_ and Fe^3+^ 2p_3/2_, respectively. The binding energies at 722.6 and 724.6 eV correspond to Fe^2+^ 2p_1/2_ and Fe^3+^ 2p_1/2_, respectively. The binding energies at 730.0 and 732.3 eV are satellite peaks of Fe^2+^ 2p_1/2_ and Fe^3+^ 2p_1/2_, respectively.

**Table 4:** Peak area and position of Fe in prehnite samples

Name	Position (eV)	Area							
		Oxidizing atmosphere				Reducing atmosphere			
		OA200-F	OA400-F	OA600-F	OA800-F	RA200-F	RA400-F	RA600-F	RA800-F
Fe^2+^ 2p_3/2_	709.0	4060.24	3398.35	2227.28	1835.73	4544.92	4187.08	4435.88	2978.30
Fe^3+^ 2p_3/2_	711.0	7661.02	7883.70	6018.42	5248.09	6731.24	6042.51	5491.36	2897.78
Fe^2+^ sat 2p_3/2_	715.6	3045.62	3222.20	2639.12	2215.27	3584.81	3085.83	3626.03	1993.35
Fe^3+^ sat 2p_3/2_	718.8	1230.04	1315.63	956.11	1607.35	1083.66	1001.25	1812.81	1948.36
Fe^2+^ 2p_1/2_	722.6	2030.12	1699.18	1113.64	917.87	2272.46	2093.54	2217.94	1489.15
Fe^3+^ 2p_1/2_	724.6	3830.51	3941.85	3009.21	2624.05	3365.62	3021.26	2745.68	1448.89
Fe^2+^ sat 2p_1/2_	730.0	3474.08	3394.43	2765.31	2733.16	2660.76	2470.67	3605.70	3068.31
Fe^3+^ sat 2p_1/2_	732.3	2348.59	3108.55	1904.03	2509.42	2595.96	1500.25	2906.18	2774.53
Fe^2+^/Fe^3+^	—	0.53	0.43	0.37	0.35	0.68	0.69	0.81	1.03

Table 4 shows the peak areas of different binding energy positions for all samples. The ratio of the sum of the peak areas of Fe^2+^ 2p_3/2_ and Fe^2+^ 2p_1/2_ to the sum of the peak areas of Fe^3+^ 2p_3/2_ and Fe^3+^ 2p_1/2_ is used as the relative content ratio of Fe^2+^ to Fe^3+^, so as to determine the change of Fe^2+^ and Fe^3+^ content. The results show that with the increase of temperature, the value of Fe^2+^/Fe^3+^ gradually decreases in the oxidizing atmosphere, while it gradually increases in the reducing atmosphere (Table 4). With the increase of temperature in oxidizing atmosphere, the increase of Fe^3+^ content increases the yellow tone of the samples, while the increase of Fe^2+^ content in reducing atmosphere increase the white tone of the samples. The change of Fe^3+^ and Fe^2+^ content is also a reason for the color change of prehnite during heating.

## 4. Conclusions

In summary, the composition, structure and discoloration mechanism of the prehnite samples before and after the heat treatment were investigated. The results indicate that with the increase of temperature, part of Fe^2+^ is oxidized to Fe^3+^ in the oxidizing atmosphere, making Fe^3+^ appear in the lattice channel, while part of Fe^3+^ is reduced to Fe^2+^ in the reducing atmosphere, making Fe^2+^ exist in the octahedral position. The change of the relative content and occupation position of Fe^2+^ and Fe^3+^ in the samples will change the Raman characteristic peaks of samples in the range of 358–360 cm^−1^ caused by metal cation vibration and octahedral vibration. In oxidizing atmosphere, the increase of Fe^3+^ content in the samples will also strengthen the infrared absorption peak at 814 cm^−1^ caused by the bending vibration of H–O–Fe^3+^. The change of Fe^3+^ and Fe^2+^ content and occupancy position influence the charge transfer of Fe^2+^–Fe^3+^. When the heat treatment temperature is 200, 400 and 600 °C, the change of Fe^3+^ and Fe^2+^ content and occupation position is the main reason for the color change of prehnite samples. When the heat treatment temperature is 800 °C, sample OA800-J and sample RA800-J are partially decomposed into anorthite and wollastonite. The high temperature decomposition reaction affects the charge transfer of Fe^2+^–Fe^3+^, and also affects the color change of sample OA800-J and sample RA800-J. The obvious color change between sample OA800-J and sample RA800-J is caused by the change of relative content and occupation position of Fe^3+^ and Fe^2+^ and the high-temperature decomposition reaction. This study provides the mechanism of the color change of prehnite after heat treatment and the suitable heat treatment temperature range of prehnite.

## Conflicts of interest

There are no conflicts to declare.

## Supplementary Material
